# Association of CAPN10 SNPs and Haplotypes with Polycystic Ovary Syndrome among South Indian Women

**DOI:** 10.1371/journal.pone.0032192

**Published:** 2012-02-23

**Authors:** Shilpi Dasgupta, Pisapati V. S. Sirisha, Kudugunti Neelaveni, Katragadda Anuradha, B. Mohan Reddy

**Affiliations:** 1 Molecular Anthropology Group, Biological Anthropology Unit, Indian Statistical Institute, Hyderabad, Andhra Pradesh, India; 2 Department of Endocrinology, Osmania General Hospital, Hyderabad, Andhra Pradesh, India; 3 Anu Test Tube Baby Centre, Hyderabad, Andhra Pradesh, India; Sudbury Regional Hospital, Canada

## Abstract

Polycystic Ovary Syndrome (PCOS) is known to be characterized by metabolic disorder in which hyperinsulinemia and peripheral insulin resistance are central features. Given the physiological overlap between PCOS and type-2 diabetes (T2DM), and calpain 10 gene (CAPN10) being a strong candidate for T2DM, a number of studies have analyzed CAPN10 SNPs among PCOS women yielding contradictory results. Our study is first of its kind to investigate the association pattern of CAPN10 polymorphisms (UCSNP-44, 43, 56, 19 and 63) with PCOS among Indian women. 250 PCOS cases and 299 controls from Southern India were recruited for this study. Allele and genotype frequencies of the SNPs were determined and compared between the cases and controls. Results show significant association of UCSNP-44 genotype *CC* with PCOS (p = 0.007) with highly significant odds ratio when compared to TC (OR = 2.51, p = 0.003, 95% CI = 1.37–4.61) as well as TT (OR = 1.94, p = 0.016, 95% CI = 1.13–3.34). While the haplotype carrying the SNP-44 and SNP-19 variants (21121) exhibited a 2 fold increase in the risk for PCOS (OR = 2.37, p = 0.03), the haplotype containing SNP-56 and SNP-19 variants (11221) seems to have a protective role against PCOS (OR = 0.20, p = 0.004). Our results support the earlier evidence for a possible role of UCSNP-44 of the CAPN10 gene in the manifestation of PCOS.

## Introduction

Polycystic ovary syndrome (PCOS) is the most common reproductive endocrinopathy of women in their childbearing years and is responsible for an estimated 70% of cases of anovulatory infertility. In addition to the clinical features of hyperandrogenism and chronic anovulation, many women are insulin resistant and at increased risk for type-2 diabetes mellitus (T2DM) [1]. Previous studies have established that the prevalence of impaired glucose tolerance and T2DM among women with PCOS has been constantly increasing with consistency across populations of varied ethnic and racial backgrounds [1], [2], [3]. Genetic studies have revealed that PCOS and T2DM could share genetic susceptibility factors associated with both the pathologies. Using this hypothesis, several studies have suggested that genes related to T2DM may also play a role in PCOS pathogenesis [4]–[11]. Calpain 10 (CAPN10) is a candidate gene for T2DM, positionally cloned on 2q chromosome [12], and found to be associated with T2DM in several populations [12]–[14]. Most of the subsequent studies found association of CAPN10 gene with PCOS phenotypes as well [7]–[11], [15]. While Ehrmann *et al.* [7], Gonzalez *et al.* [8], [9] and Vollmert *et al.* [15] reported association of CAPN10 with PCOS and quantitative measures such as fasting insulin, blood glucose levels related to T2DM, Escobar-Morreale *et al.* [10] reported an association of CAPN10 polymorphism with hirsutism. In contrast, Haddad *et al.* [11] reported no association of CAPN10 with PCOS.

The nature of association of CAPN10 with PCOS has not been hitherto studied among the Asian populations in general and particularly among the Indians albeit its association is fairly established with T2DM among the Asians, including the Indian populations [16]–[18]. We present here the results of our pioneering effort in investigating the association of five CAPN10 SNPs (UCSNP-44, UCSNP-43, UCSNP-56, UCSNP-19 and UCSNP-63) with polycystic ovary syndrome among South Indian women.

## Results

### Clinical characteristics of the study population

For majority of the PCOS cases, the data on clinical and/or biochemical parameters were obtained and the characteristics that are relevant to the metabolic component of PCOS are presented in Table 1, according to the BMI categories. The total number of cases ‘N’ denotes the number of cases for which the respective data could be obtained. Over 90% of the PCOS cases were aged below 30 and only one woman aged above 35. The random blood sugar (RBS) as well as fasting insulin (FI) levels of these cases were in the normal range, hence, non-diabetic. Therefore, no further tests related to diabetic profile were warranted. In the present study, the recruitment was made only after confirmation of PCOS through the Rotterdam criteria. Most of the patients approached the collaborating clinicians with the problem of menstrual irregularities, who were subsequently diagnosed as PCOS after confirmation of their ultrasonographic and/or hyperandrogenic status. Therefore, the data presented is not a mere presentation of the prevalence of the diagnostic features, but the actual data of the patients that were encountered in the clinical centers. All the cases with ultrasound data had polycystic ovary morphology along with the clinical presentation of irregular menstrual cycles. Nearly 79% of the PCOS cases in the present study were reported to be infertile and were under treatment. The remaining patients were young unmarried women primarily approaching the clinician with the symptom of irregular menses before being diagnosed as PCOS, hence their infertility status could not be ascertained. Among the other clinical features of PCOS, hirsutism and acanthosis nigricans were significantly more frequent in the obese PCOS cases than the lean PCOS cases (p<0.001). A significantly greater proportion of obese cases had elevated cholesterol levels (>200 mg/dl) than the lean PCOS cases (p = 0.002). Unfortunately, the biochemical parameters could not be obtained for the control group which would have enabled a comparison between the cases and controls.

**Table 1:** Clinical profile of the PCOS cases under study.

Phenotypic features	All patients		Lean PCOS (BMI<25)		Obese PCOS (BMI≥25)**	
	N	%	N	%	N	%
PCO	124/124	100	30/30	100	43/43	100
Menstrual irregularities	210/210	100	56/56	100	104/104	100
Hirsutism*	175/230	76.1	41/54	75.9	96/101	95
Acne	47/230	20.4	13/54	24.1	23/101	22.7
Acanthosis nigricans*	110/220	50	19/54	35.2	68/101	67.3
Infertility	181/230	78.7	43/55	78.2	77/104	74
Elevated Cholesterol (>200 mg/dl)*	38/150	25.3	1/25	4	25/80	31.2

* significantly different between lean PCOS and obese PCOS cases (p≤0.002).

** Given the lean body mass of Indian women (Asian Indian Phenotype), BMI≥25 is generally considered as cut off distinguishing the lean women from overweight/obese women albeit the WHO criteria specifies BMI>30 as obese.

### Genotype and allele frequencies of CAPN10 SNPs

The genotype and allele frequency distribution for the five SNPs are presented in Table 2 and Table 3, respectively. We observed a significantly higher frequency of homozygotes for the polymorphic variant C at the UCSNP-44 locus (intron 3) among the PCOS cases than the controls (17.4% vs 8% respectively, p = 0.007). This trend was also seen in the allele distribution pattern, wherein the variant allele C was found in 27.8% of the PCOS cases as compared to 22.4% of the controls. However, the statistical significance for this allele was not retained after Bonferroni correction for multiple testing. Logistic regression analysis of PCOS status on the UCSNP-44 genotypes taking age and BMI as covariates yielded significant odds ratio for the *CC* genotype when compared to *CT* or *TT* genotype (p = 0.003, p = 0.016 respectively) (Table 4) with a statistical power (1-β error probability) of 99%.

**Table 2:** Genotype frequency distribution of CAPN10 polymorphisms among PCOS cases and controls.

SNP ID	UCSNP ID	Gene location	Genotype	PCOS Cases (N = 248)	Controls (N = 298)	*α^2^ (p-value)
rs2975760	44	Intron 3	TT	0.618	0.640	9.79 (0.007)
			TC	0.207	0.271	
			CC	0.174	0.089	
rs3792267	43	Intron 3	GG	0.828	0.824	2.93 (0.23)
			GA	0.159	0.127	
			AA	0.012	0.031	
rs2975762	56	Intron 4	GG	0.292	0.269	2.33 (0.31)
			GA	0.483	0.447	
			AA	0.224	0.284	
rs3842570	19	Intron 6	Del/Del#	0.229	0.228	0.09 (0.96)
			Del/Ins#	0.459	0.449	
			Ins/Ins#	0.310	0.322	
rs5030952	63	3′UTR	CC	0.916	0.913	0.88 (0.65)
			CT	0.076	0.070	
			TT	0.008	0.017	

* Standard Pearson chi square value with degree of freedom = 2.

# Del – Deletion.

Ins – Insertion.

**Table 3:** Allele frequency distribution of CAPN10 polymorphisms among PCOS cases and controls.

SNP ID	UCSNP ID	Allele	PCOS Cases (2 N = 496)	Controls (2 N = 596)	* *α* ^2^ (p-value)
rs2975760	44	T	0.722	0.775	4.08 (0.04)
		C	0.278	0.224	
rs3792267	43	G	0.908	0.905	0.02 (0.89)
		A	0.092	0.094	
rs2975762	56	G	0.534	0.493	1.72 (0.18)
		A	0.466	0.507	
rs3842570	19	Del	0.540	0.547	0.05 (0.83)
		Ins	0.460	0.453	
rs5030952	63	C	0.954	0.948	0.19 (0.65)
		T	0.046	0.052	

* Standard Pearson chi square value with degree of freedom = 1.

**Table 4:** Logistic regression analysis of UCSNP-44 for association with PCOS, taking age and BMI as covariates.

SNP ID	UCSNP	Genotypes compared	Odds ratio	95% CI@	p-value
rs2975760	UCSNP-44	CC vs TT	1.94	1.13–3.34	0.016
		CC vs CT	2.51	1.37–4.61	0.003
		CC/CT vs TT	1.05	0.73–1.50	0.784

@ CI – Confidence interval.

The case cohort was also analyzed in two groups, based on body mass index (BMI), i.e. lean PCOS (BMI<25) and obese PCOS (BMI≥25) cases. The allele and genotype frequencies were compared between these two groups as well as each of them with controls (pooled). The control group was also categorized according to BMI, and similar comparison was carried out between the BMI matched case and control groups (lean cases vs lean controls, and obese cases vs obese controls). This analysis did not yield any significant observation (results not presented).

Using PyPop software, the observed genotype counts were compared with those expected under Hardy–Weinberg proportions (HWPs), and a χ^2^ test was carried out to check for the significance of deviation from HWP for each SNP locus in PCOS women and controls separately. All the loci, except UCSNP-44, confirm to the Hardy-Weinberg equilibrium, in both the cases and the controls. The departures from HWP, in case of UCSNP-44, were observed to be due to increased proportion of the homozygotes in both cases and controls.

Given that our sample consisted of sizeable cohort of Muslim subjects, we repeated the genotype analysis for the Hindu caste and Muslim subjects separately. But this categorization does not seem to change genotype distribution profiles to any significant degree (results not presented). Since the Muslim and Hindu cases were predominantly drawn from the Osmania General Hospital and Anu Test Tube Baby Centre, respectively, which represented lower and higher socioeconomic strata, the results also do not suggest any effect of socioeconomic status in the pattern of manifestation of PCOS in relation to CAPN10 SNPs. Further, we drew 50, 60 & 70% random subsets of case and control samples and repeated the above analysis on each of the subsets to test for internal consistency. Overall, the results suggest that the pattern of distribution of the CAPN10 alleles and their association with PCOS in each of the subsets is similar to the total sample (results not presented) indicating internal consistency.

### Linkage Disequilibrium among CAPN10 SNPs and Haplotype analysis

**Figure 1:**
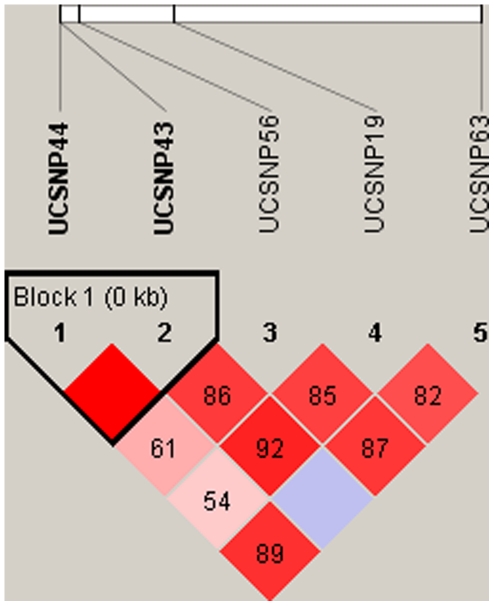
Linkage disequilibrium plot for CAPN10 polymorphisms in the entire cohort of PCOS cases and controls. D′ values are mentioned in the LD blocks.

Out of 250 PCOS cases and 299 controls, 223 cases and 266 controls could be used for the purpose of estimating linkage disequilibrium (LD) for the five CAPN10 SNPs. As per the LD plot (Figure 1), only two loci (UCSNP-44 and UCSNP-43) were in perfect LD (D′ = 1). Except the loci combinations of SNP-44:SNP56, SNP-44:SNP-19 and SNP-43:SNP-63, all the other combinations exhibited strong LD (D′ = 0.82–0.92). Pairwise LD estimates were also made for the case and control groups separately (Table 5).While UCSNP-43 and UCSNP-44 were in perfect LD (D′ = 1) in both the groups, UCSNP-63 depicted different LD patterns among the cases and controls. While in cases it was observed to be in perfect LD with UCSNP-56 and UCSNP-19, among the controls, UCSNP-63 shows strong LD with both UCSNP-43 and -44 (D′ = 1). Strong LD was also observed for UCSNP-19:UCSNP-43, UCSNP-19:UCSNP-56 and UCSNP-43:UCSNP56 in both case and control groups.

**Table 5:** Pairwise LD estimates in PCOS cases and controls. Data are D′ values.

LOCUS PAIR	PCOS CASES (N = 223)	CONTROLS (N = 266)	TOTAL COHORT (N = 489)
UCSNP19 : UCSNP43	0.91475	0.93013	0.92
UCSNP19 : UCSNP44	0.54032	0.55093	0.54
UCSNP19 : UCSNP56	0.90743	0.80621	0.85
UCSNP19 : UCSNP63	1	0.74352	0.82
UCSNP43 : UCSNP44	1	1	1
UCSNP43 : UCSNP56	0.91568	0.83889	0.86
UCSNP43 : UCSNP63	0.02091	1	0.00
UCSNP44 : UCSNP56	0.53209	0.71771	0.61
UCSNP44 : UCSNP63	0.8095	1	0.89
UCSNP56 : UCSNP63	1	0.797	0.87

Haplotypes based on the five CAPN10 SNPs (UCSNP-44, -43, -56, -19 and -63) were constructed and analyzed for possible association with PCOS (Table 6). A total of nine haplotypes, with a frequency of >1%, was obtained in the entire cohort of 489 individuals. Interestingly, we could infer both risk conferring and protective haplotypes from their comparative distribution in the case and control groups. The proportion of 21121 and 11221 haplotypes differed significantly between the case and control groups. While 21121 haplotype was significantly more frequent among the cases, the 11221 haplotype was overrepresented among the controls. The logistic regression analysis using 11121 as reference haplotype suggests significant odds for both 21121 and 11221 haplotypes (Table 7), albeit in different directions; while 21121 is risk conferring, 11221 is protective against PCOS. Two other haplotypes (21221 and 11111) also exhibited marginal association with the control group. However, after performing Bonferroni correction for multiple testing, the association pattern remained significant only for 11221 haplotype.

**Table 6:** CAPN10 haplotype frequency distribution among PCOS cases and controls.

Haplotype	PCOS cases (2 N = 446)	Controls (2 N = 532)	*α* ^2^ *	p-value
21211	0.211363	0.170812	2.66	0.103
21221	0.008581	0.026489	4.07	0.043
21121	0.062426	0.030489	5.97	0.014
11211	0.190294	0.199673	0.21	0.645
11212	0.041883	0.046397	0.14	0.705
11221	0.015426	0.061543	14.27	<0.0001
11111	0.012502	0.032079	4.25	0.039
11121	0.353587	0.327145	0.73	0.392
12121	0.085226	0.081487	0.01	0.903

* Standard Pearson chi square value with degree of freedom = 1.

**Table 7:** CAPN10 haplotype effect: Haplotypic OR by comparison to the reference with its 95% CI.

Haplotype	Odds Ratio*	95% CI	p- value
21121	2.37	1.06–5.30	0.035
11221	0.20	0.06–0.59	0.004

* (in comparison to the reference haplotype 11121).

### Analysis of specific haplogenotypes with clinical traits in PCOS

**Figure 2:**
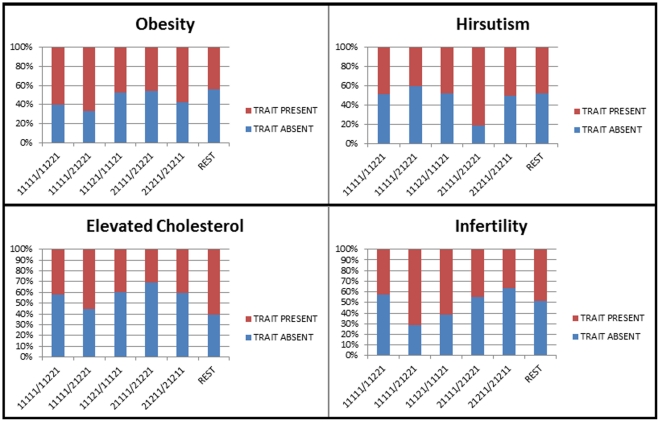
Analysis of specific CAPN10 haplotypes with different clinical phenotypes of PCOS.

Given the significant haplotype association results, we further explored the role of these specific haplotypes in the manifestation of certain clinical characteristics of PCOS. We compared different haplotype combinations between two groups of patients divided on the basis of presence/absence of a particular PCOS phenotypic trait (i.e hirsutism, obesity, infertility, and hypercholesterolemia) (Figure 2). While a greater proportion of the hirsutism trait was observed in cases with 21111/21221 combination, all the other traits i.e. obesity, infertility and elevated cholesterol levels (>200 mg/dl) were found in greater frequency among cases with 11111/21221 combination. Although these comparisons provided some pattern of association between the haplotype combinations and phenotypic traits, the magnitude of differences between the two groups of patients was not statistically significant.

## Discussion

PCOS, as a syndrome, has multiple components- reproductive, metabolic and cardiovascular- with long term health implications. In addition to the clinical features of hyperandrogenism and chronic anovulation, many PCOS women are insulin resistant and are at high risk to develop type-2 diabetes. Clinical and laboratory based studies in PCOS have variously pointed to abnormalities of insulin receptor binding, or more plausibly, post receptor signaling as well as to the evidence for a primary abnormality of insulin secretion [19]. Therefore, numerous genes involved in insulin action and secretion have been explored as candidate genes in the PCOS pathology. The CAPN10 gene, encoding a ubiquitous member of the calpain-like cysteine protease family, plays a role in insulin secretion and action [20] and was positionally cloned within the NIDDM1 region [12]. The presence of calpain-10 mRNA in pancreatic islets, muscle, and liver, the three most important tissues that control blood glucose levels, suggests that calpain-10 may regulate pathways that affect insulin secretion, insulin action, and hepatic glucose production, each of which is altered in patients with type 2 diabetes [20]. Variation in the calpain-10 gene was reported to be linked and associated with type 2 diabetes mellitus (T2DM) susceptibility in a Mexican American population [12]. Specific combinations of three intronic variants, designated as “SNP- 43,” “SNP-19,” and “SNP-63,” that capture most of the haplotype diversity at CAPN10 were associated with a three-fold increased risk for T2DM in this population [12]. However, Evans *et al.* [13] reported that another variant, SNP-44 located in intron 3 and separated by 11 base-pairs from SNP-43, was independently associated with T2DM in whites from the United Kingdom. Apart from being a strong candidate for T2DM [12], [13], the CAPN10 gene has been widely evaluated in traits such as PCOS and idiopathic hirsutism [7]–[11], [15] due to the fact that PCOS and type-2 diabetes share a number of etiologic factors. Preliminary studies of CAPN10 gene in PCOS patients from the United Kingdom provide the first evidence of CAPN10 involvement in PCOS, suggesting a statistically significant association between the UCSNP-44 allele and PCOS susceptibility (cited in Gonzalez *et al.* [9]). Subsequent studies evaluating the role of CAPN10 in PCOS have yielded contradictory results. Supporting the CAPN10 gene involvement in PCOS, Gonzalez *et al.* [8], [9] showed that CAPN10 UCSNP-44 allele was associated with PCOS in the Spanish population. Another study in the Spanish population has suggested that CAPN10 UCSNP-43 allele somehow influences the hirsutism score in hyperandrogenic patients while UCSNP-45 could be associated with idiopathic hirsutism [10]. While Ehrmann *et al.* [7] and Vollmert *et al.* [15] have shown association of UCSNPs-19, -63 and UCSNPs-19, -56, respectively, with PCOS phenotype, Haddad *et al.* [11] did not find any association between CAPN10 SNPs and PCOS. The significance of the association between the SNPs/haplotypes with PCOS is underlined by the positive correlation of two adjacent SNPs - UCSNP-43 and UCSNP-44 - specifically with hyperandrogenic features that are central to PCOS [9], [10].

Some recent CAPN10 studies among Indian populations have focused on T2DM, but not on PCOS [16]–[18]. While Cassell *et al.* [16] and Adak *et al.* [17] reported that haplotypes containing SNP-19 and SNP-63 alleles increase risk for T2DM, Bodhini *et al.* [18] concluded that 2111 haplotype of SNPs –44, –43, –19, and –63 may be associated with type 2 diabetes mellitus, although none of these SNPs may be individually associated with diabetes. In an attempt to explore possible association of CAPN10 gene variants with risk for PCOS, we carried out this study in a largest cohort of South Indian women with PCOS hitherto examined for CAPN10 SNPs in a case-control setup. Even with a minimum effect size of 0.1, our sample size of 549 (250 cases+299 controls) is large enough and far exceeds the estimated number of samples (∼250 cases+controls) required to obtain a 90% statistical power.

We selected a panel of five SNPs in the CAPN10 gene which was based on the knowledge of their previous association with PCOS as well as T2DM [7]–[18]. While UCSNP-43 alone has been strongly associated with T2DM and insulin resistance, both UCSNP-43 and -44 have been implicated in the transcriptional regulation of the CAPN10 gene [12]. Of the five SNPs analyzed, we found significant association of UCSNP-44 with PCOS at the genotype as well as allele frequency level. The homozygous genotype for the polymorphic allele of UCSNP-44 (*CC*) was associated with 2–2.5 fold increase in risk for PCOS as compared to the other genotypes. Given the internal consistency of our findings and their concurrence to the earlier evidence [8], [9], the present study underscores the importance of UCSNP-44 of CAPN10 in PCOS pathophysiology. The significance of this association is further highlighted through the functional studies suggesting that SNP-44 is located in an enhancer element and might affect CAPN10 expression [12].

In our cohort, the haplotype 21121 (UCSNPs -**44**,-43,-56,-**19** and -63) was found to be significantly associated with PCOS (p = 0.014) although this significance was not retained after Bonferroni correction; this haplotype, comprising of the variant alleles of UCSNP-44 and UCSNP-19, exhibited a two-fold increased risk of PCOS (OR = 2.37, p = 0.03). We also obtained significant association of haplotype 11221 (UCSNPs -44,-43,-**56**,-**19** and -63), comprising of UCSNP-56 and UCSNP-19 variant alleles, with increased frequency among the controls, which was significant even after Bonferroni correction (p<0.0001). We could infer from the odds ratio (OR = 0.20, p = 0.004) that this haplotype (11221) might have a significant protective role against PCOS. However, analysed individually, neither of the variants for UCSNP-56 and UCSNP-19 depicted any significant difference in genotype or allele frequency distribution patterns. We find both risk conferring and protective haplotypes in our cohort, with UCSNP-19 variant allele being common to both of them. This could probably be due to relatively large minor allele frequency of UCSNP-19 (45%) in our cohort. This has reflected in 11121 being the most frequent haplotype comprising only UCSNP-19 as the variant allele among the PCOS cases and controls with a frequency of 35% and 33% respectively. Our haplotype results are partially concurrent to the observations of Bodhini *et al.* [17] concerning the presence of UCSNP-44 allele in the risk conferring haplotypes in another South Indian sample, suggesting UCSNP-44 as an important marker in the pathophysiology of PCOS and/or T2DM particularly among South Indian populations. The difference in the risk conferring haplotypes between the two studies lies at the UCSNP-19 locus (211**2**1 versus 21-**1**1), even though the variant allele frequency for this locus is comparable between the two samples(45% in our study; 56% in the earlier study). Further, compared to Bodhini *et al.* [17] UCSNP-56 locus is additionally studied by us. The variant allele frequencies of all the CAPN10 SNPs were found to be homogenous among different Indian populations as compared to the Chinese, European and Mexican-American populations (Table 8).

**Table 8:** Minor allele frequencies of CAPN10 markers in different populations.

Genetic Marker	China^a^	USA^a^	Mexican Americ-ans^a^	Finland^a^	Germany		UK^c^	Spain^d^	India			
					i^a^	ii^b^			i^e^	ii^f^	ii^g^	iii^h^
UCSNP-44	–	–	0.06	–	–	0.15	0.15	0.11	0.19	0.21	–	0.22
UCSNP-43	0.06	0.19	0.27	0.32	0.29	0.31	0.25	0.24	0.14	0.02	0.19	0.09
UCSNP-56	–	–	–	–	–	0.38	–	–	–	–	–	0.50
UCSNP-19	0.38	0.40	0.41	0.28	0.29	0.37	0.39	0.34	0.56	0.30	0.40	0.45
UCSNP-63	0.31	0.06	0.23	0.03	0.04	0.06	0.07	0.08	0.03	0.05	0.02	0.05

a Horikawa *et al.* (2000).

b Vollmert *et al.* (2007).

c Evans *et al.* (2001).

d Gonzalez *et al.* (2004).

e Cassell *et al.* (2002).

f Bodhini *et al.* (2010).

g Adak *et al.* (2010).

h Present study.

Given the significant association of certain haplotypes, we analyzed the pattern of haplotype distribution vis-à-vis the different clinical phenotypes of PCOS. Irrespective of the association pattern, we observed two haplotype combinations: while 21111/21221 was more prominent among the PCOS cases with hirsutism, the other combination (11111/21221) showed a relatively greater frequency among the cases with infertility, obesity and elevated cholesterol levels, suggesting variable effect of UCSNP-44 depending on its presence in homozygous/heterozygous state. Overall, the results of our pioneering study among the Indian women are concurrent to the earlier observations that emphasize the role of CAPN10 UCSNP-44 in the manifestation of PCOS. Nevertheless, further studies are warranted to replicate the association patterns in larger cohorts of ethnically diverse populations of India so as to reach unequivocal conclusion on the role of CAPN10 polymorphisms in PCOS. Functional studies based on the regulatory regions of this gene would be required further to help gain more meaningful further insights on the precise etiological role of CAPN10 towards PCOS phenotype.

## Materials and Methods

### Study design

A total of 549 women consisting of 250 PCOS cases (aged 14–40 years) and 299 controls (aged 14–47 years) were enrolled for the present study from July 2008 to April 2009. Patients were recruited from the Gynecology clinic of the Osmania General Hospital, Hyderabad as well as from an infertility clinic (Anu's Test Tube Baby Centre, Hyderabad) as per the Rotterdam criteria, 2003 (The Rotterdam ESHRE/ASRM-sponsored PCOS Consensus Workshop Group, 2004) [21] according to which any two of the following three conditions need to be fulfilled for the inclusion: (i) presence of clinical and/or biochemical signs of hyperandrogenism, (ii) infrequent periods with intermenstrual interval of more than 35 days, and (iii) polycystic ovaries; an ovary with the ultrasound appearance of more than 10 subcapsular follicles (<10 mm in diameter) in the presence of prominent ovarian stroma was considered polycystic. Patients with hyperprolactinemia, thyroid and adrenal diseases, 21-hydroxylase deficiency, and androgen-secreting tumors were excluded. The weight and height of the subjects were recorded. Hirsutism was defined as a Ferriman-Gallwey score of more than 5 [22]. Hormonal assays that were recorded included serum levels of gonadotrophic hormones {luteinizing hormone (LH) and follicle stimulating hormone (FSH)}, thyroid stimulating hormone (TSH), prolactin, testosterone (total), fasting insulin (FI) and random blood sugar (RBS) levels. Normal controls with no history of treatment for fertility, no evidence of clinical hyperandrogenism (hirsutism/acne/alopecia), and with normal menstrual cycles every 25–32 days were recruited from the family planning center of the Osmania hospital and from the general population.

### Ethics Statement

Intravenous blood samples (∼5 ml) were collected from both the patients and controls after obtaining their informed written consent. The study protocol was approved by the Indian Statistical Institute Review Committee for Protection of Research Risks to Humans.

### DNA extraction, amplification and sequencing

DNA was extracted from the peripheral blood samples of the patients and controls using the phenol-chloroform method [23]. We carried out PCR amplification and sequencing to screen the CAPN10 polymorphisms using the forward and reverse primers. Each PCR was optimized with respect to the concentration of Mg2+ ions. The PCR-mix consisted of 10×PCRBuffer, 10 µM dNTP-mix, 1 µM of each primer, 1 U Taq-polymerase and 40 ng template DNA in a reaction volume of 10 µl. Reactions were carried out in an ABI GeneAmp9700 thermal cycler (Applied Biosystems, Foster City, CA). Forward and reverse primers and annealing temperature are given in Table 9.

**Table 9:** Primers used for CAPN10 polymorphism genotyping.

UCSNP ID	Primers (5′→3′)		Conditions (35 cycles)		Product size (bp)
	Forward primer	Reverse primer	Annealing	Extension	
UCSNP-44	GCTGGCTGGTGACATCAGTG	TCAGGTTCCATCTTTGGGCCAG	59°C – 30″	72°C – 30″	475
UCSNP-43	GCTGGCTGGTGACATCAGTG	TCAGGTTCCATCTTTGGGCCAG	59°C – 30″	72°C – 30″	475
UCSNP-56	AGGCCTCAGGCACACTGTAG	AGACAGTGGGCTTTGACTCG	58°C – 30″	72°C – 30″	98
UCSNP-19	GTTTGGTTCTCTTCAGCGTGGAG	CATGAACCCTGGCAGGGTCTAAG	60°C – 30″	72°C – 30″	155/187
UCSNP-63	GAACCAGTGCTTGGCAGCTCAC	GCAGTGCGTGGTGCCTGAAGG	58°C – 30″	68°C – 45″	476

Cycle Sequencing of PCR products were carried out with either the forward or the reverse primers using the Big-Dye Terminator ready reaction kit (Applied Biosystems, Foster City, CA). Extended products were purified by ethanol precipitation and analyzed on an ABI 3730 automated DNA Analyzer (Applied Biosystems, Foster City, CA). No new data has been generated through our sequencing work. Since only the known polymorphisms of CAPN10 gene have been sequenced, the respective rsIDs are provided in the tables.

### Statistical Analysis

All the statistical analyses were performed with the help of SPSS statistical software (version 19.0, IBM SPSS). Power of the study was calculated using G*Power software (version 3.1). The Hardy-Weinberg equilibrium was estimated by the χ^2^ test using Pypop software. Haploview and THESIAS softwares were used to estimate LD and generate haplotype frequencies. For all tests, significance level was set as p<0.05

## References

[1] Ehrmann DA, Barnes RB, Rosenfield RL, Cavaghan MK, Imperial J (1999). Prevalence of impaired glucose tolerance and diabetes in women with polycystic ovary syndrome.. Diabetes Care.

[2] Legro RS, Kunselman AR, Dodson WC, Dunaif A (1999). Prevalence and predictors of risk for type 2 diabetes mellitus and impaired glucose tolerance in polycystic ovary syndrome: a prospective, controlled study in 254 affected women.. J Clin Endocrinol Metab.

[3] Ehrmann DA, Chang RJ, Heindel JJ, Dunaif A (2002). Glucose Intolerance in Polycystic Ovary Syndrome: Role of the Beta Cell.. Polycystic Ovary Syndrome.

[4] Urbanek M, Legro RS, Driscoll DA, Azziz R, Ehrmann DA (1999). Thirty-seven candidate genes for polycystic ovary syndrome: strongest evidence for linkage is with follistatin.. Proc Natl Acad Sci.

[5] Villuendas G, Escobar-Morreale HF, Tosi F, Sancho J, Moghetti P (2003). Association between the D19S884 marker at the insulin receptor gene locus and polycystic ovary syndrome.. Fertil Steril.

[6] El Mkadem SA, Lautier C, Macari F, Molinari N, Lefe'bvre P (2001). Role of allelic variants Gly972Arg of IRS-1 and Gly1057Asp of IRS-2 in moderate-to severe insulin resistance of women with polycystic ovary syndrome.. Diabetes.

[7] Ehrmann DA, Schwarz PE, Hara M, Tang X, Horikawa Y (2002). Relationship of calpain-10 genotype to phenotypic features of polycystic ovary syndrome.. J Clin Endocrinol Metab.

[8] Gonzalez A, Abril E, Roca A, Aragon MJ, Figueroa MJ (2002). CAPN10 alleles are associated with polycystic ovary syndrome.. J Clin Endocrinol Metab.

[9] Gonzalez A, Abril E, Roca A, Aragon MJ, Figueroa MJ (2003). Specific CAPN10 gene haplotypes influence the clinical profile of polycystic ovary patients.. J Clin Endocrinol Metab.

[10] Escobar-Morreale HF, Peral B, Villuendas G, Calvo RM, Sancho J (2002). Common single nucleotide polymorphisms in intron 3 of the calpain-10 gene influence hirsutism.. Fertil Steril.

[11] Haddad L, Evans JC, Gharani N, Robertson C, Rush K (2002). Variation within the type 2 diabetes susceptibility gene calpain-10 and polycystic ovary syndrome.. J Clin Endocrinol Metab.

[12] Horikawa Y, Oda N, Cox NJ, Li X, Orho-Melander M (2000). Genetic variation in the gene encoding calpain-10 is associated with type 2 diabetes mellitus.. Nat Genet.

[13] Evans JC, Frayling TM, Cassell PG, Saker PJ, Hitman GA (2001). Studies of association between the gene for calpain-10 and type 2 diabetes mellitus in the United Kingdom.. Am J Hum Genet.

[14] Weedon MN, Schwarz PE, Horikawa Y, Iwasaki N, Illig T (2003). Meta-analysis and a large association study confirm a role for calpain-10 variation in type 2 diabetes susceptibility.. Am J Hum Genet.

[15] Vollmert C, Hahn S, Lamina C, Huth C, Kolz M (2007). Calpain-10 variants and haplotypes are associated with polycystic ovary syndrome in Caucasians.. Am J Physiol Endocrinol Metab.

[16] Cassell PG, Jackson AE, North BV, Evans JC, Syndercombe-Court D (2002). Haplotype combinations of calpain 10 gene polymorphisms associate with increased risk of impaired glucose tolerance and type 2 diabetes in South Indians.. Diabetes.

[17] Bodhini D, Radha V, Ghosh S, Sanapala KR, Majumder PP (2010). Association of calpain 10 gene polymorphisms with type 2 diabetes mellitus in Southern Indians.. Metab Clin Exp.

[18] Adak S, Sengupta S, Chowdhury S, Bhattacharyya M (2010). Co-existence of risk and protective haplotypes of Calpain 10 gene to type 2 diabetes in the eastern Indian population.. Diab & Vasc Disease Res.

[19] Franks S, Gharani N, McCarthy M (2001). Candidate genes in polycystic ovary syndrome.. Hum Reprod Update.

[20] Sreenan SK, Zhou YP, Otani K, Hansen PA, Currie KPM (2001). Calpains play a role in insulin secretion and action.. Diabetes.

[21] The Rotterdam ESHRE/ASRM-sponsored PCOS Concensus Workshop Group (2004). Revised 2003 Concensus on diagnostic criteria and long term health risk related to Polycystic Ovary Syndrome.. Fertil Steril.

[22] Mifsud A, Ramirez S, Yong EL (2000). Androgen receptor gene CAG trinucleotide repeats in anovulatory infertility and polycystic ovaries.. J Clin Endocrinol Metab.

[23] Sambrook J, Fritschi EF, Maniatis T (1989). Molecular cloning: a laboratory manual.

